# The role of hydrophobic matching on transmembrane helix packing in cells

**DOI:** 10.15698/cst2017.11.111

**Published:** 2017-11-02

**Authors:** Brayan Grau, Matti Javanainen, Maria Jesús García-Murria, Waldemar Kulig, Ilpo Vattulainen, Ismael Mingarro, Luis Martínez-Gil

**Affiliations:** 1 Departamento de Bioquímica y Biología Molecular, ERI BioTecMed, Universitat de València, E-46100 Burjassot, Spain.; 2 Laboratory of Physics, Tampere University of Technology, FI-33101 Tampere, Finland.; 3 Department of Physics, University of Helsinki, POB 64, FI-00014 Helsinki, Finland.; 4 MEMPHYS - Centre for Biomembrane Physics.

**Keywords:** hydrophobic match, mismatch, Glycophorin A, membrane protein folding, helix packing, transmembrane domain dimerization

## Abstract

Folding and packing of membrane proteins are highly influenced by the lipidic component of the membrane. Here, we explore how the hydrophobic mismatch (the difference between the hydrophobic span of a transmembrane protein region and the hydrophobic thickness of the lipid membrane around the protein) influences transmembrane helix packing in a cellular environment. Using a ToxRED assay in *Escherichia coli* and a Bimolecular Fluorescent Complementation approach in human-derived cells complemented by atomistic molecular dynamics simulations we analyzed the dimerization of Glycophorin A derived transmembrane segments. We concluded that, biological membranes can accommodate transmembrane homo-dimers with a wide range of hydrophobic lengths. Hydrophobic mismatch and its effects on dimerization are found to be considerably weaker than those previously observed in model membranes, or under *in vitro* conditions, indicating that biological membranes (particularly eukaryotic membranes) can adapt to structural deformations through compensatory mechanisms that emerge from their complex structure and composition to alleviate membrane stress. Results based on atomistic simulations support this view, as they revealed that Glycophorin A dimers remain stable, despite of poor hydrophobic match, using mechanisms based on dimer tilting or local membrane thickness perturbations. Furthermore, hetero-dimers with large length disparity between their monomers are also tolerated in cells, and the conclusions that one can draw are essentially similar to those found with homo-dimers. However, large differences between transmembrane helices length hinder the monomer/dimer equilibrium, confirming that, the hydrophobic mismatch has, nonetheless, biologically relevant effects on helix packing *in vivo*.

## INTRODUCTION

Assembly of the native structure of most integral membrane proteins takes place in two main steps 1. The first step includes targeting and insertion of the protein into a lipid membrane. In the case of alpha-helical membrane proteins this initial step occurs generally co-translationally (coupled with the translation of the protein) through the translocon, a multiprotein complex that facilitates not only the insertion of integral membrane proteins into the lipid bilayer but also translocation of soluble proteins into the endoplasmatic reticulum (ER) lumen 2. In the second stage, if required, the transmembrane (TM) segments interact to form the tertiary and quaternary structure of the mature functional membrane protein.

While for water-soluble proteins the dynamics and energetics underlying their folding have been studied thoroughly, the extent of similar studies in the context of membrane proteins is much more modest. This is quite surprising, given that membrane proteins represent about 30% of the human proteome 3,4,5 and are involved in a significant fraction of key cellular processes such as signaling, energy production, etc. Due to their different environments, the forces that underlie the folding process are also distinct for water- and membrane-soluble proteins 6,7,8. For water-soluble proteins, the folding is largely driven by hydrophobic interactions. In the folding of membrane proteins, the role of the hydrophobic effect is less relevant and applies mainly to the formation of secondary structures 9. Also, while salt bridges and aromatic interactions are important in the folding of water-soluble proteins, they do not contribute significantly to membrane protein folding 10. Meanwhile, there are forces such as inter-helical hydrogen bonding and especially van der Waals interactions that have only a minor role in the folding of soluble proteins, while they are considered major driving forces in protein folding within lipid bilayers 7,8.

One of the means used by membranes to control TM domain conformation is hydrophobic matching, i.e, the matching between the hydrophobic span of a TM segment and the hydrophobic thickness of the lipid membrane around the protein 11,12. Given that exposure of hydrophobic groups in proteins and lipids to water is highly unfavorable, membranes tend to minimize their free energy by maximizing the matching between the hydrophobic length of the bilayer and the TM helices. However, in some cases, there is a disparity thus creating a hydrophobic mismatch (positive when the (hydrophobic) TM segment is longer than the hydrophobic thickness of membrane, and negative when the membrane non-polar region is thicker than the hydrophobic region of the peptide 13). The resulting energetic penalty is thought to be compensated either by membrane or peptide rearrangements, including TM segment packing 13. Intriguingly, while this concept has been explored quite extensively for individual TM domains (peptides) in model membranes and also under *in vitro* conditions 14,15, it has received much less attention in the more realistic setting of living cells.

Glycophorin A (GpA) represents one of the best suited and most studied models for alpha-helical TM segment packing and membrane protein folding 16,17. GpA homo-dimerization relies exclusively on its unique TM domain 18. The sequence motif within the TM segment driving the association can be reduced to five residues, namely G79VxxGVxxT87 (where x represents any hydrophobic residue) 19. Amidst this motif, the glycine residues play a crucial role. Their disposition, coupled with the tilt of the helix, renders close packing of two monomers, thereby maximizing significant interactions between the TM segments 20. However, experimental results have shown that, at least *in vitro*, the formation of GpA dimers is not solely dependent on the protein sequence. The lipid environment can also make a significant contribution 21. It has been shown that not only the lipid composition but also the hydrophobic mismatch between the GpA TM segment and the surrounding hydrophobic environment of the lipid membrane can modify the monomer-dimer equilibrium 19. The above view based on experimental work is supported by molecular simulations of model systems, where GpA has served as a centerpiece. Hence, Molecular Dynamics (MD) simulations on GpA have been employed to investigate phenomena such as membrane insertion 22, dimer structure 23,24, and dimerization energetics 25.

Since its introduction in the nineties 26, the concept of hydrophobic mismatch has received, extensive attention both experimentally (*in vitro*) 27,28,29,30 and computationally 31,32,33. However, as we mentioned above, the implications of hydrophobic matching on membrane protein folding, packing, and oligomerization have not been investigated in biological membranes of cells. In the present manuscript, we explore hydrophobic matching and its effects through GpA dimerization in prokaryotic and eukaryotic cells. To this end, we utilized an *Escherichia coli* fluorescence-based assay (ToxRED system) and a Bimolecular Fluorescent Complementation (BiFC) assay in mammalian cells. We show that even in an *in vivo* scenario with a complex membrane system, the hydrophobic matching does contribute to GpA dimerization, i.e., quaternary structure assembly. However, the significance of this effect in biological membranes is much weaker than under *in vitro* conditions, suggesting that biological membranes are far more adaptable to this stress than previously thought. This conclusion is backed up by extensive atomistic simulations in a number of GpA-lipid membrane systems with varying peptide lengths and membrane thicknesses. These simulations provide an atom-scale interpretation of the mechanisms used by lipid membranes to stabilize dimers exposed to hydrophobic mismatch. To our knowledge, this is the first systematic *in vivo* study of hydrophobic matching, providing a key step for a better understanding of membrane protein folding under native conditions.

## RESULTS

### Packing of TM segments with different length in biological membranes

To assess the influence of hydrophobic matching on the packing of TM segments, one should vary the length of either the TM segments or the model membrane system employed in the assay. Working in cell, the only modifiable variable is the length of the TM segment used. For this purpose, we constructed a series of chimeric stretches bearing the minimal dimerization domain found in GpA (GVxxGVxxT, where x represents an hydrophobic residue) 19 with increasing number of leucines forming TM segments (**Table 1**). All the hydrophobic regions designed were identified as potential TM segments by the ΔG prediction server 34 (**Table 1**). Likewise, multiple TM protein prediction algorithms classified all the resultant chimeric proteins as membrane proteins. The designed TM segments range from 17 to 29 residues long. The rise per residue along the axis in a canonical helix is 1.5Å. Therefore, a stretch of approximately 20 consecutive hydrophobic amino acids will span 30Å of the hydrocarbon core of a biological membrane. Indeed, the most prevalent length of TM helices is 21 residue 35. By selecting TM segments that are either longer or shorter than 21 residues we can induce a discrepancy in the membrane thickness that allows us to investigate the role of this imbalance in the TM segment packing.

**Table 1:**
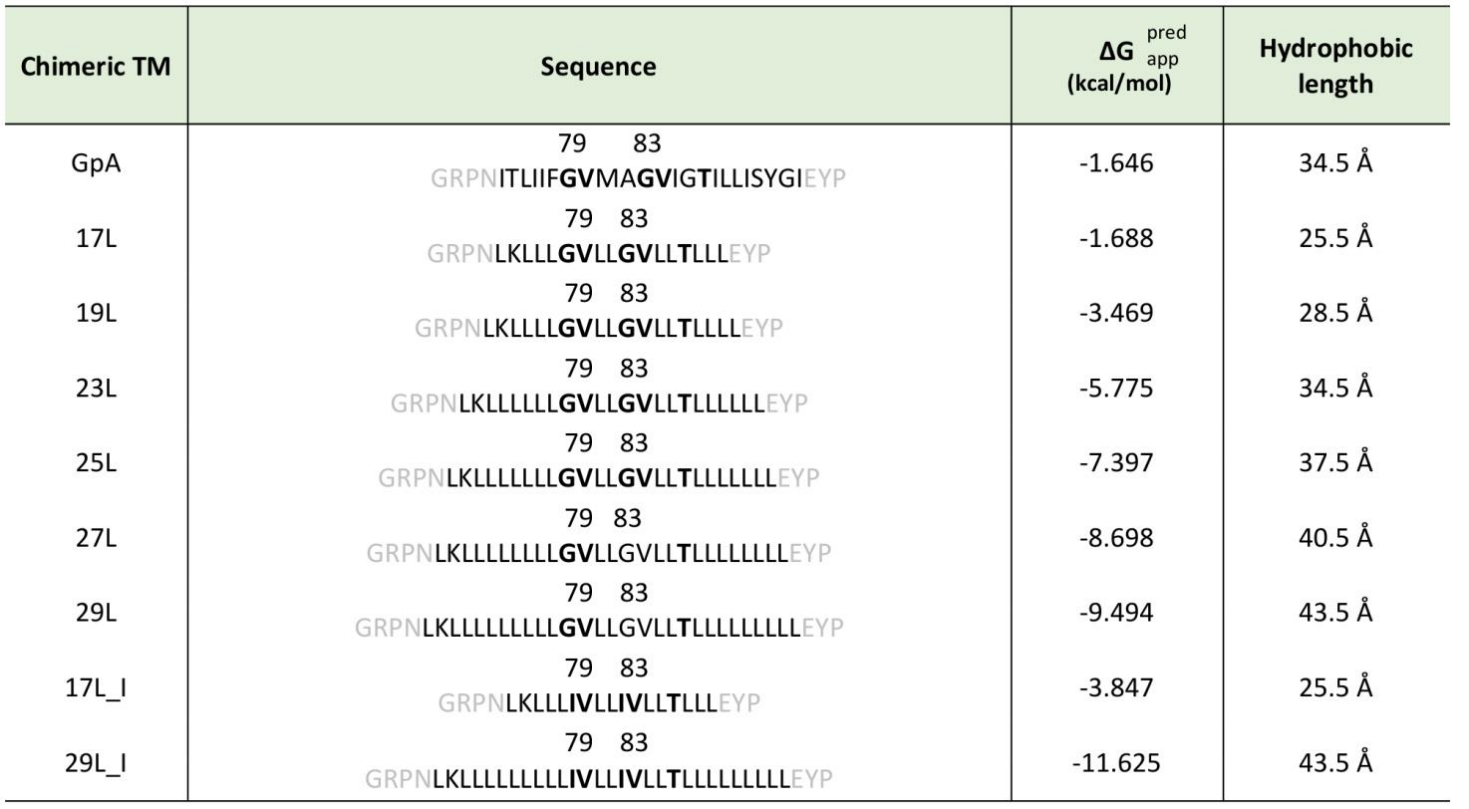
Table 1. Sequences predicted ΔG and hydrophobic length of transmembrane segments. Chimeric TM segments were named based on their hydrophobic length (amino acids). The sequence of each TM segment is included. The dimerization motive is highlighted in bold (including the amino acid position in the wild-type GpA sequence) and flanking regions are indicated in gray. The apparent predicted ΔG for the insertion of the hydrophobic regions (calculated by the ΔG prediction server v1.0, where negative values are indicative of insertion) and the hydrophobic length (calculated assuming 1.5 Å per residue in a α-helix conformation) were also included in the table.

**Figure 1:**
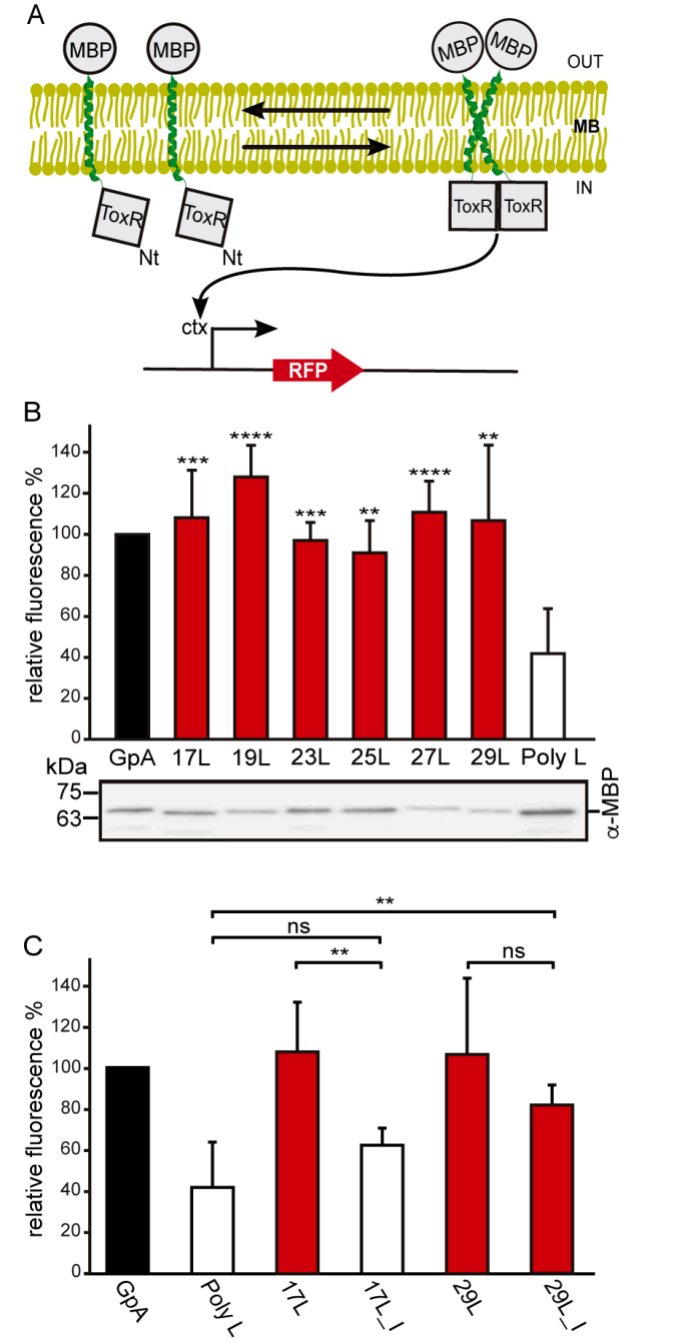
FIGURE 1: Homo-dimerization in *E. coli* membranes. **(A)** Schematic representation of ToxRED Assay. TM-driven oligomerization results in dimerization of ToxR transcriptional activator which, ultimately, drives the expression of the red fluorescence protein RFP (encoded under *ctx* promoter). The c-terminus maltose binding protein (MBP) located in the periplasm (OUT) allows growth of *E. coli* MM39 strain in M9 minimal media supplemented with 0.8% of maltose. **(B)** Mean relative fluorescence of chimera homo-oligomerization. Error bars denote standard deviation obtained from at least 6 independent experiments (p-values for the comparison with poly L: ** <0.01, *** <0.001, **** <0.0001). The color intensity-code was used to highlight dimerization (red) vs non-dimerization (white). The positive control (GpA homo-dimer) is shown in black. The α-MBP western blot under the bar graph shows chimera’s expression levels. **(C)** Contribution of Gly for the dimerization of TM chimeras. Mean relative fluorescence of 17L, 29L, 17L_I, and 29I_L chimeras homo-oligomerization. Error bars denote standard deviation obtained from at least 4 independent experiments (p-values for the comparison with poly L: ** <0.01, ns non-significant). The color intensity-code is used to highlight dimerization (red) vs non-dimerization (white). Positive control (GpA homo-dimer) is highlighted in black.

In order to understand the hydrophobic matching effect in a cellular environment we first studied the ability of the aforementioned TM segments to homo-dimerize in *E. coli* membranes. To this end, we utilized a variation of the ToxCAT assay 36 known as ToxRED 37. Briefly, this methodology uses a chimeric construct composed of the N-terminus DNA binding domain of ToxR (a dimerization-dependent transcriptional activator), fused to the challenged TM segment and a periplasmic anchor (the maltose binding protein, MBP) needed for the growth of the bacteria in minimal media supplemented with maltose 38. Dimerization through the TM segments results in ToxRED-mediated activation of the ctx promoter which drives the synthesis of the Red Fluorescent Protein (RFP) (**Figure 1A**). RFP values were normalized using the absorbance of the bacteria culture (600 nm) to rule out culture growth differences as the source of fluorescence variations (note that in this system the growth of the MM39 *E. coli* strain depends on the proper expression and insertion of the chimeric protein). Furthermore, the correct expression of all the constructs was assessed by western blot using an anti-MBP antibody (**Figure 1B**, bottom). Our results show that, all the chimeras bearing the minimized GpA dimerization motif, despite their different hydrophobic lengths, were capable of forming homo-dimers that rendered RFP levels similar to those obtained using the wild-type GpA TM segment (a 23 hydrophobic residue long segment), and significantly higher than the negative controls (a 13 amino acid long stretch of leucines, poly L that efficiently inserts into the membranes 39,40) (**Figure 1B**). It has been shown that long TM hydrophobic segments can lead to oligomerization 41,42. To isolate the contribution of the hydrophobic length on the oligomerization of our segments we mutated the Gly residues to Ile in the 17L (17L_I) and 29L (29L_I) constructs (**Figure 1C**) (the sequence, hydrophobic length, and predicted ΔG values of these segments are included in **Table 1**). While elimination of GxxxG motif in the 17L backbone decreased the ToxRED associated fluorescence to background levels, the Gly to Ile substitutions had a minor effect on 29L, indicating that the positive mismatch can induce TM segment packing in *E. coli* membranes.

**Figure 2:**
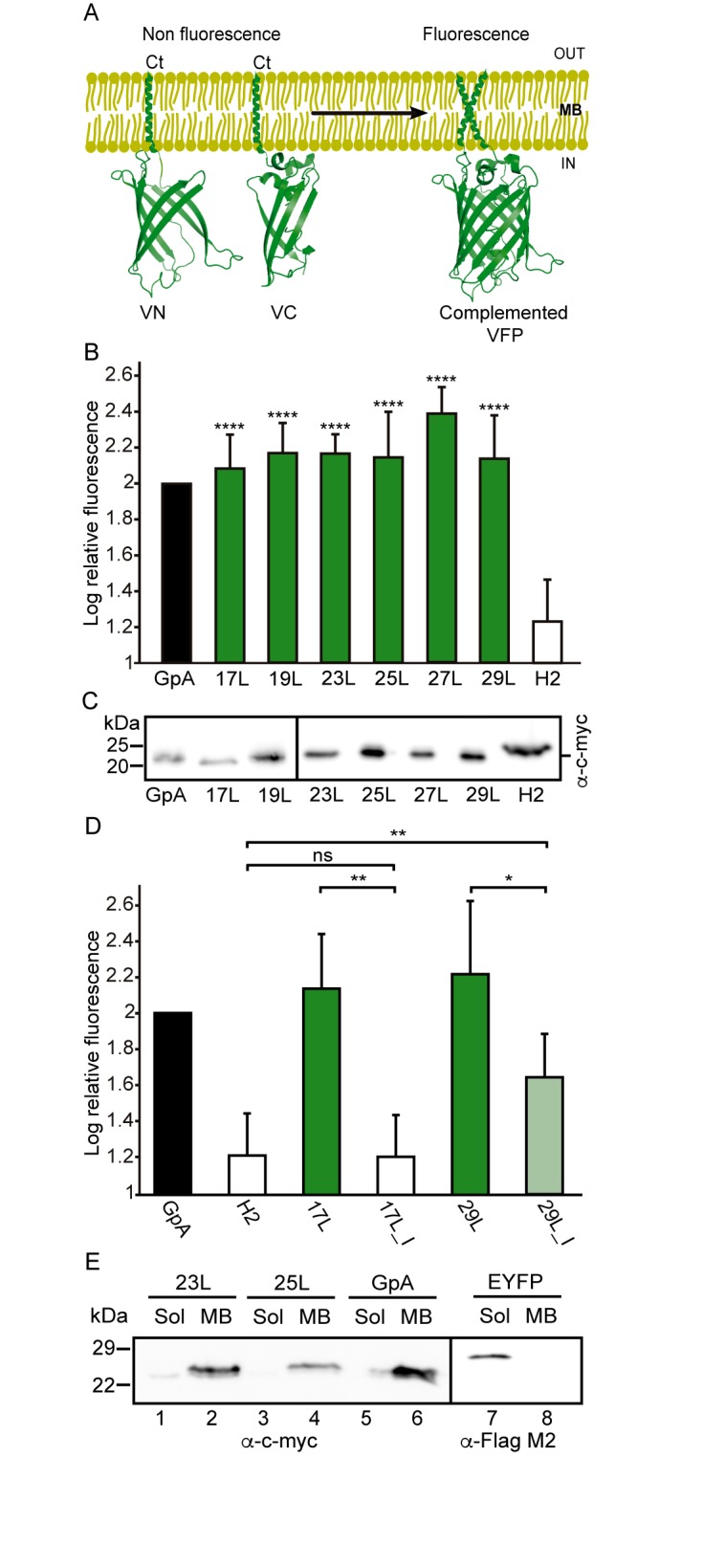
FIGURE 2: Homo-dimerization in eukaryotic membranes. **(A)** Schematic representation of BiFC Assay. TM-driven oligomerization results in complementation of two non-fluorescent halves (amino-terminus (VN) and carboxy-terminus (VC)) of the Venus Fluorescent Protein (VFP). **(B)** Mean relative fluorescence of chimera homo-oligomerization in HEK293T cells (GpA (VN-GpA/VC-GpA), 17L (VN-17L/VC-17L), 19L (VN-19L/VC-19L), 23L (VN-23L/VC-23L), 25L (VN-25L/VC-25L), 27L (VN-27L/VC-27L), 29L (VN-29L/VC-29L), H2 (VN-H2/VC-H2)). Error bars indicate standard deviation obtained from at least 4 independent replicates (H2 was used as a negative control, **** < 0.0001). A color intensity code is used to highlight dimerizing (green) and non-dimerizing (white) transmembrane segments, while positive dimerization control (GpA) is shown in black. **(C)** Western Blot show chimera’s expression levels detected by α-c-Myc antibody. **(D)** The contribution of Gly for the dimerization of TM chimeras*. *Relative fluorescence of chimera homo-oligomerization in human-derived HEK293T cells (GpA [VN-GpA/VC-GpA (depicted in black)], H2 [VN-H2/VC-H2] (white), 17L [VN-17L/VC-17L], 17L_I [VN-17L_I/VC-17L_I], 29L [VN-29L/VC-29L], 29L_I [VN-29L_I/VC-29L_I). The bars represent mean values of chimera homo-oligomerization, and error bars denote standard deviation obtained from 3 independent experiments (p-values of Student’s t-test: * < 0.05, ** < 0.01, ns (non-significant)). Color intensity is used to highlight dimerizing (significantly different from H2 control, green) and non-dimerizing (white). Light green is used to indicate those samples (where the Gly residues of the dimerization domain have been substituted with Ile) which fluorescence values are significantly higher than the H2 control and at the same time lower than the corresponding non-mutated control. **(E)** Cellular fractionation of chimera expressing eukaryotic cells*.* Subcellular fractionation of HEK293T cells expressing BiFC chimeras (c-Myc tagged) (VN-23L, VN-25L and VN-GpA) or EYFP (Flag-tagged) (used as a soluble marker). Soluble fraction (Sol) and Membrane fraction (MB).

Additionally, we analyzed the formation of homo-dimers in eukaryotic cells utilizing a BiFC assay 43. Briefly, the Venus Fluorescent Protein (VFP) was divided into two non-fluorescent parts: amino-terminus (VN) and carboxyl-terminus (VC). Each half was then fused to the hydrophobic segments previously designed (**Table 1**) and expressed in human-derived HEK293T cells as in 34. Oligomerization of the TM domains allows the reconstitution of the full-length VFP and the recovery of its fluorescence properties (**Figure 2A**). Similarly to the ToxRED assay, we included native GpA TM homo-dimers as a positive control and normalization value. As a negative control, we used the second TM segment of *E. coli* Leader peptidase (H2), a non-dimerizing protein widely used in membrane protein biogenesis studies 44. In eukaryotic membranes, we observed, once again, the strong dimerization of all the tested GpA chimeras regardless of their hydrophobic length (from 17L to 29L) (**Figure 2B**), with fluorescence values comparable to those obtained when the wild-type TM segment of GpA was used. A western blot was included to monitor protein levels (**Figure 2C**). In the BiFC assay we also investigated the contribution of the GpA dimerization domain to the interaction between TM monomers. Once again, Gly residues were substituted to Ile in the 17L and 29L constructs (VN and VC) 45. In both cases, in contrast to the ToxRED results, the elimination of the Gly residue significantly reduced the observed fluorescence (**Figure 2D**). Nonetheless, we could observe an increased fluorescence of the 29L_I as compared to the 17L_I, suggesting that positive but not negative hydrophobic mismatch can partially drive oligomerization in eukaryotic membranes. The resemblance between the BiFC and ToxRED assays suggest that biological membranes, despite their origin (eukaryotic or prokaryotic), behave similarly, but not equally, when packing TM helices.

Contrarily to the ToxRED assay, in which the ToxR and MBP moieties have to face the cytosol and the periplasm, respectively, the BiFC approach cannot discern whether the chimeras are being inserted into the membrane or remain cytosolic. To distinguish between these two possibilities we performed subcellular fractionation treatments in which the membrane and cytosolic fractions were separated (**Figure 2E**). The chimeras bearing 23L, 25L, or wild-type GpA TM segments were located in the membrane fraction (lanes 2, 4, and 6 respectively). Conversely, the EYFP (used as a soluble marker) was found exclusively in the cytosolic fraction (lane 7).

To gain more detailed insight into how cellular membranes adapt to hydrophobic mismatch, we performed atomistic molecular dynamics simulations on 17L, 23L, and 29L (containing the minimized dimerization motif) homo-dimers embedded in single-component DLPC (12:0 PC), DOPC (18:1 PC), and DEPC (22:1 PC) bilayers. This choice of bilayer systems provides a systematic increase in membrane thickness. The hydrophobic thickness values of 20.0 Å, 27.4 Å, and 35.7 Å correspond to phosphorus-phosphorus thicknesses of 31.6 Å, 38.7 Å, and 47.3 Å, respectively. The DOPC bilayer is likely the closest mimic of the membranes studied *in vivo*, as palmitic (16:0), palmitoleic (16:1), and oleic (18:1) fatty acids are the most common lipid chains in *E. coli* 46 and HEK293T 47 membranes. Together with the hydrophobic lengths of 25.5, 34.5, and 43.5 Å estimated for the 17L, 23L, and 29L peptides, respectively (**Table 1**), the different combinations allow us to consider both positive (TM hydrophobic length > membrane thickness) and negative (TM hydrophobic length < membrane thickness) mismatch. Notably, the extent of mismatch studied here is larger than in previous studies on GpA dimers 23. Furthermore, the unsaturation of the longer chains ensured that all bilayers remained in the liquid disordered phase at the physiological temperature of 37°C.

**Figure 3:**
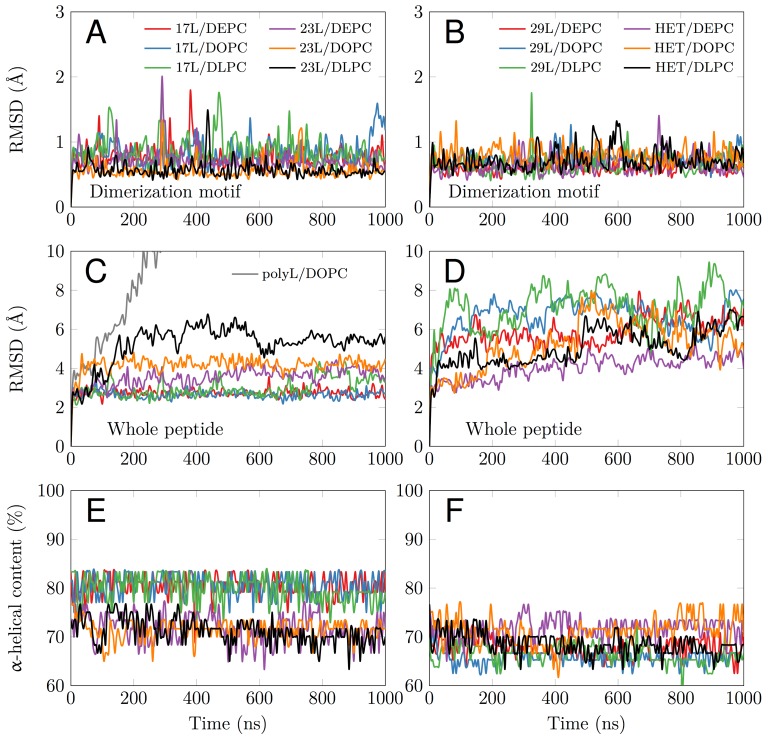
FIGURE 3: Stability of the dimeric structures from MD simulations. **(A-D)** Root mean squared deviation (RMSD) of the dimerization motif (5 residues per peptide) is shown on the top row and the RMSD of the whole dimer on the bottom row. The whole trajectory is included in the analyses. The 17L/29L hetero-dimer is labeled "HET", and the polyleucine control in DOPC as "PolyL/DOPC". **(E and F)** Helicity of the peptides during the full simulation time period (color code as in At to D). The 17L/29L hetero-dimer is labeled "HET".

**Figure 4:**
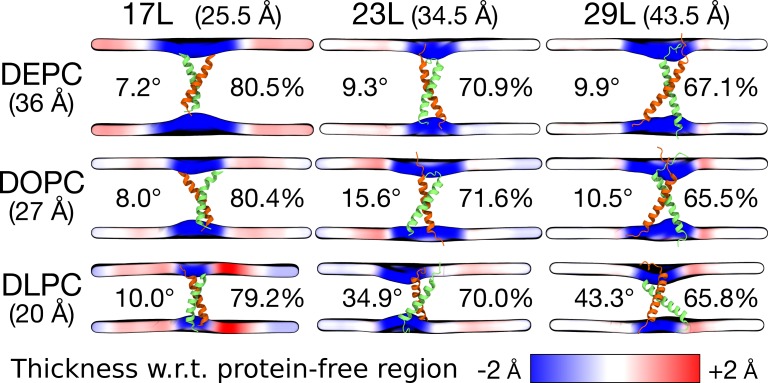
FIGURE 4: Summary of the results from MD simulations of homo-dimers. The peptide and the membrane are unchanged along the columns and rows, respectively. The hydrophobic length of the peptide and the hydrophobic thickness of the membrane are given in brackets. The two peptides are shown in green and orange, while the mean positions of the phosphorus atoms in lipid head-groups are shown as a surface. This surface is colored according to the value of local average membrane (phosphorus-phosphorus) thickness with respect to (w.r.t.) the bulk membrane thickness far away from the dimer. The thicknesses are calculated from the last 500 ns of the simulations. The average tilt of the peptides is given in degrees and the average alpha-helical content in percentage.

All GpA-based dimers maintained their transmembrane positioning in the membrane (see Figure S1) and were stable during the 1 μs simulations. This is evidenced by the time evolution of the RMSD of the dimerization motif (defined as GVxxGVxxT) shown in **Figure 3A** and **B**, which suggests that this region is equally stable regardless of the mismatch. This observation corroborates the experimental findings described above, showing that all GpA chimeras containing the minimized dimerization motif were capable to form stable homo-dimers. As shown in **Figure 3C**, the polyleucine (lacking the dimerization motif) dimer dissolved rapidly in a DOPC membrane. The RMSD of all the rest GpA-based dimers reveals that the shortest 17L dimers were overall very stable due to their location within the membrane (**Figure 3A** and **3C**), while the ends of the longest 29L dimers were more mobile since they reside in the aqueous phase (Figure S2). The stability of the 23L dimers decreases upon increasing membrane thickness as their ends become more exposed to water and therefore more prone for unfolding. The increase in the fluctuations within the termini region due to increasing mismatch (negative to positive) was also evident in the RMSF data shown in Figure S2, and explained by a lower overall alpha-helical content of the peptides (see **Figure 3E** and **F** and **Figure 4**). Interestingly, the alpha-helical content of the peptides in a given dimer is independent of the thickness of the host membrane. However, when placed in the same membrane, the longer the peptides are, the lower their alpha-helical content is. Still, although the percentages of the alpha-helical content decrease as the peptides get longer, the absolute number of the amino acids in the alpha-helical conformation actually increases. Thus, some but not all added Leu residues (17L→23L→29L), originally in a helical conformation, unfold rapidly during the short equilibration simulations, during which position restraints are turned off.

**Figure 5:**
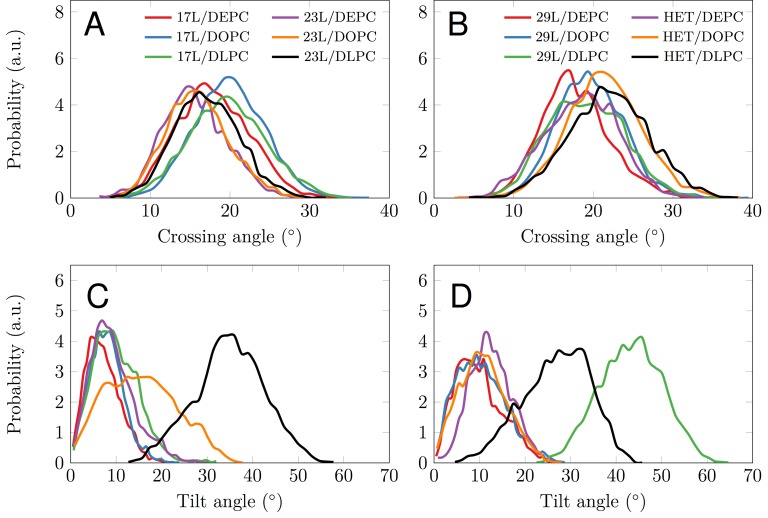
FIGURE 5: Tilt and crossing angle distribution. **(A, B)** Crossing angle distribution of the dimers calculated from the last 500 ns of the simulations. The 17L/29L hetero-dimer is labeled "HET". **(C-D)** Tilt angle distribution of the dimers calculated from the last 500 ns of the simulations. The 17L/29L hetero-dimer is labeled "HET".

Despite the varying levels of mismatch, the stability of the dimers suggests that either the peptide dimer or the membrane is able to compensate for effects induced by the mismatch. The compensation likely takes place through structural adjustment. In the case of positive mismatch, the tilt angle of the dimer shown in **Figure 4** and **Figure 5** reveals that the collective tilting of the dimer plays a role only in the case of the longer peptides (23L or 29L) simulated in the thinnest DLPC membrane, where the mismatch is larger, causing the dimer to tilt significantly (~40°) maintaining the crossing angle between monomers. In other cases, the dimer stands almost upright in the membrane (average tilt angle being <20°). As shown in **Figure 3E** and **F**, the peptides also maintain their alpha-helical content throughout the production simulation, and no stretching to the 310 helix structure is observed. Meanwhile, in the case of a negative mismatch, the tilt angle remains low (**Figure 4** and **Figure 5C**). Notably, there is little adaptation to the hydrophobic mismatch by a scissor-like motion of the dimers, as seen in the peptide crossing angles plots (**Figure 5A** and **B**).

For both positive and negative mismatch, the membrane thickness was perturbed only locally as demonstrated in **Figure 4**. The spatial extent of the perturbation depends on the level of mismatch, though. For the 17L dimer, the effect was smallest in the case of DLPC membrane and increases systematically with increasing bilayer thickness (DLPC<DOPC<DEPC). For the 23L and 29L dimers, the smallest perturbation was observed in the case of DEPC membrane. The effect became more prominent in the DOPC membrane, which is thinner than DEPC, however, the effect did not increase further in the DLPC membrane as the tilting of the dimers began to dominate the membrane organization. Notably, the differences in the behavior of the studied peptides in the DOPC membrane, whose thickness resembles that of the bilayers studied here *in vivo*, are insignificant. All the described adaptation mechanisms can be observed in the movie available here.

### Influence of transmembrane hydrophobic length mismatch on heterotypic helix-helix packing

**Figure 6:**
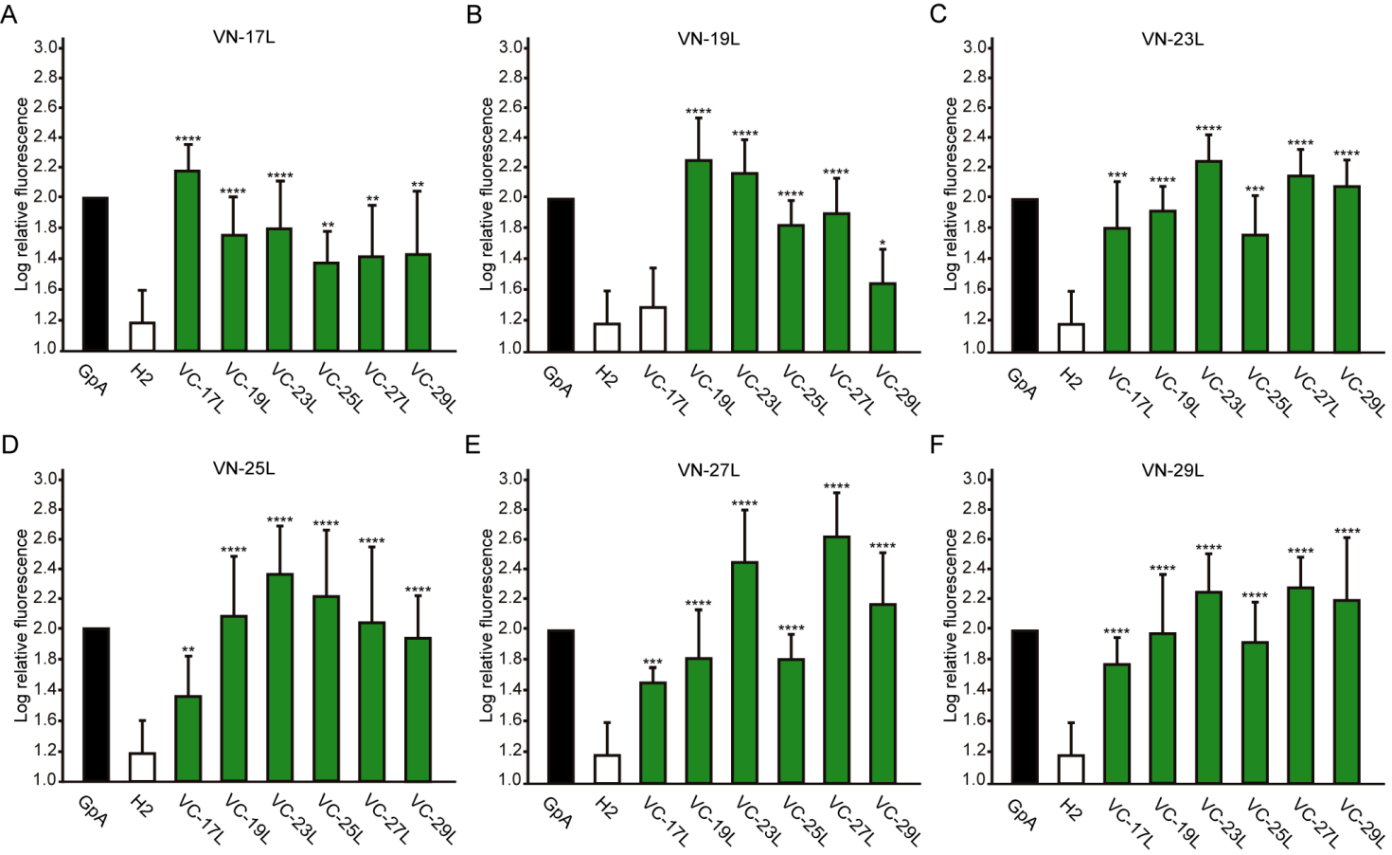
FIGURE 6: Hetero-dimerization in eukaryotic membranes. Mean relative fluorescence of chimera hetero-oligomerization in HEK293T cells of all different combinations: **(A)** VN-17L/VC-X, **(B)** VN-19L/VC-X, **(C)** VN-23L/VC-X, **(D)** VN-25L/VC-X, **(E)** VN-27L/VC-X, **(F)** VN-29L/VC-X. Error bars indicate standard deviation obtained from at least 4 independent replicates. GpA homo-dimer (black bars) was used as positive control and normalization value while Lep H2 homo-oligomer was used as a negative control (white bars). For the experimental samples, a color intensity code was used to highlight dimerization (green, fluorescence values significantly higher than those obtained with the H2 control) and non-dimerization (white, values equivalents to those obtained with the negative control). Additionally asterisks were included to indicate the level of significance (**<0.01, ***< 0.001, **** < 0.0001).

**Figure 7:**
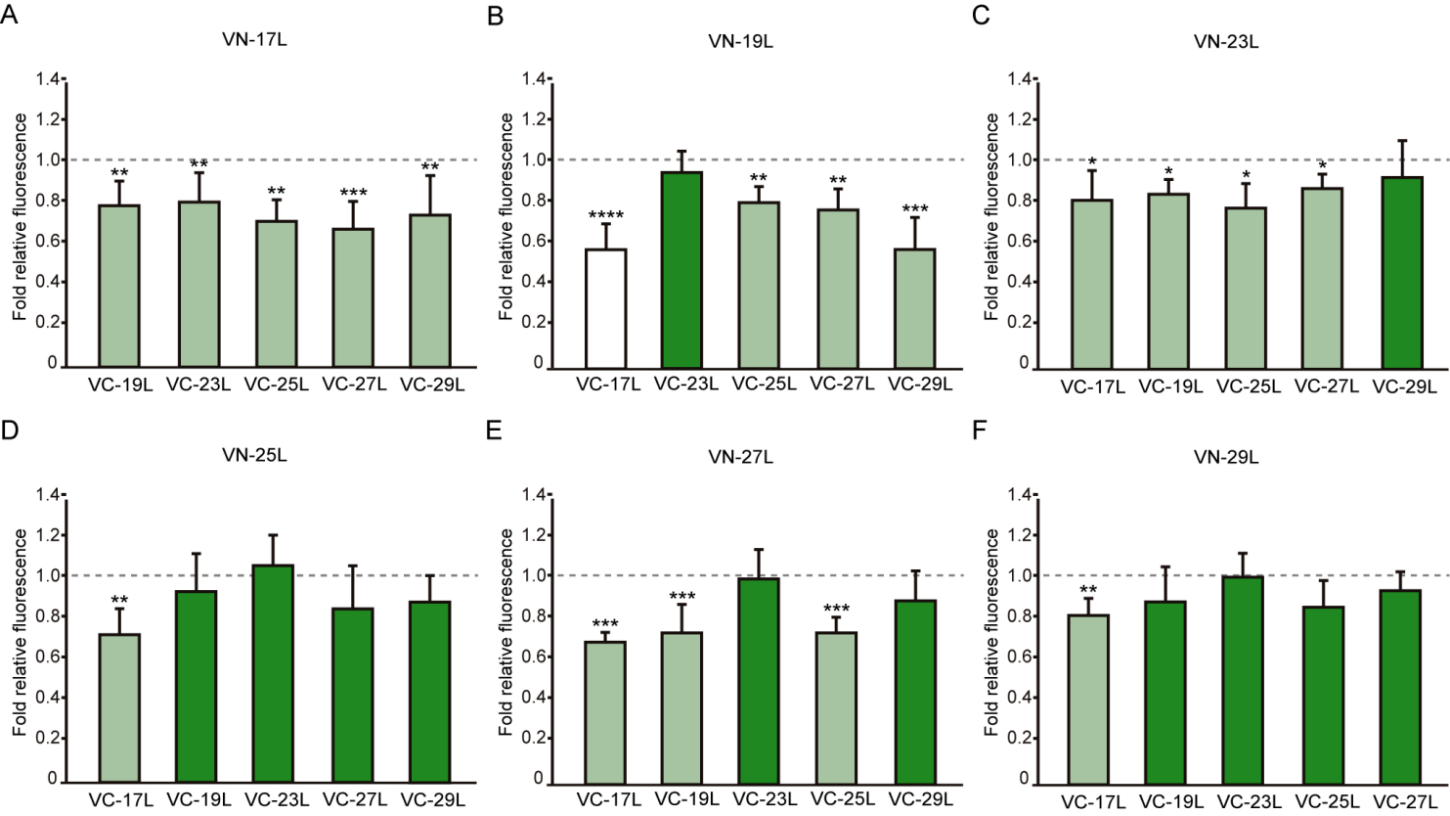
FIGURE 7: Differences in hetero-dimerization in eukaryotic membranes. Mean relative fluorescence of chimera hetero-oligomerization. For any given combination (eg.VN-X/VC-Y) the corresponding homo-oligomerization values (VN-X/VC-X and VN-Y/VC-Y) were used as a reference set to obtain fold change and significance (q-values,*<0.05, **<0.01, ***< 0.001, **** <0.0001). **(A)** VN-17L/VC-X, **(B)** VN-19L/VC-X, **(C)** VN-23L/VC-X, **(D)** VN-25L/VC-X, **(E)** VN-27L/VC-X, **(F)** VN-29L/VC-X. Error bars indicate the standard deviation obtained from at least 4 independent replicates. A color intensity code was used to highlight the dimerization intensity. Dark green (fluorescence values equivalent to the appropriated control), light green (fluorescence values significantly higher than those obtained with the H2 control but significantly lower than those observed with the corresponding homo-oligomer controls), and white (values equivalent to those obtained with the H2 control).

Next, we analyzed the potential hetero-dimer formation between TM segments with different hydrophobic lengths. The GpA homo-dimer was used as a positive reference value set while the H2 was used as a negative control. Surprisingly, the majority of the tested combinations between VN- and VC- GpA-derived chimeras (all but the VN-19L/VC-17L) were capable of reconstituting the VFP and produced fluorescence values significantly higher than the negative control (H2) (**Figure 6**). To highlight differences among the tested combinations we re-analyzed our data using the values of the homo-dimers as a reference set. In this case, for any given combination (e.g., VN-X/VC-Y) the corresponding homo-oligomerization values (VN-X/VC-X and VN-Y/VC-Y) were merged and used to obtain the fluorescence fold change unit (**Figure 7**). Light green bars indicate that the dimerization value for the selected heteromeric combination is significantly lower than its homo-dimer reference set (the level of significance is depicted with the corresponding number of asterisks on top of each bar). Contrarily dark green bars show those heteromeric combinations in which the VFP fluorescence was as high as the appropriated homomeric reference set. The VN-19L/VC-17L was depicted in white to indicate that not only the fluorescence was lower than in its controls but also not statistically higher than in the non-dimerizing H2 control. Collectively, our data suggest that a different hydrophobic length between the monomers hinders the formation of the dimer. Furthermore, a difficulty in hetero-dimer formation in biological membranes can be observed either when a large disparity between the hydrophobic length of the monomers is found or when one of the GpA-derived chimeras contains a hydrophobic region below ~28 Å (17L). Heat map representations of the data in **Figure 6** and **7** data are included in Figure S3A and S3B.

**Figure 8:**
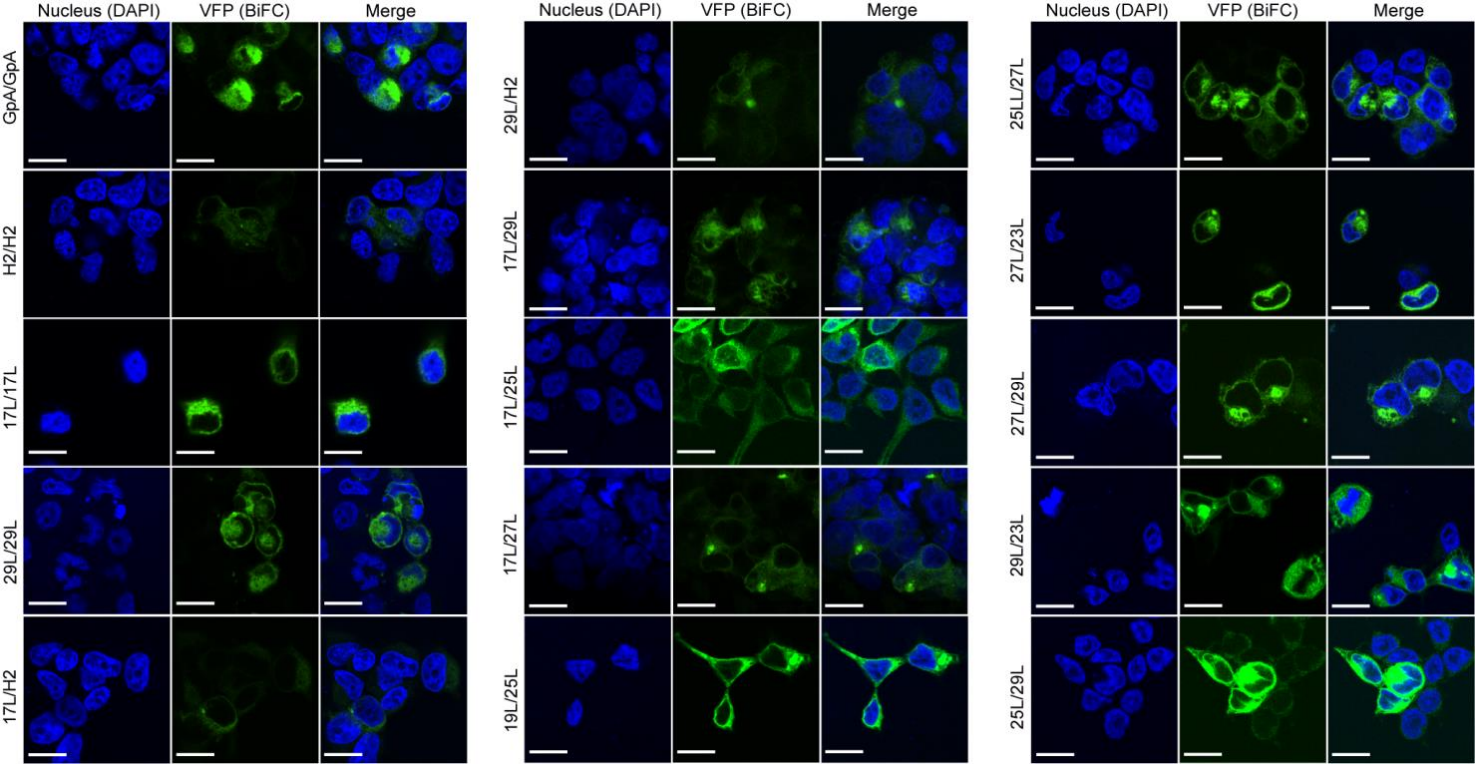
FIGURE 8: Confocal microscopy analysis for membrane dimer formation. Confocal microscopy of DAPI stained (blue) HEK293T cells expressing representative VN-VC combinations (GpA [VN-GpA/VC-GpA], 17L [VN-17L/VC-17L], 29L [VN-29L/VC-29L], 17L/H2 [VN-17L/VC-H2], 29L/H2 [VN-29L/VC-H2], 17L/29L [VN-17L/VC-29L]). Successful TM-driven oligomerization results in VFP reconstitution and fluorescent signal (green). Scale bar size is 16 μm.

The aforementioned results were corroborated by confocal microscopy (**Figure 8**). To this end, we selected the VN-17L/VC-H2 and VN-29L/VC-H2, and the VN-GpA/VC-GpA combinations as negative and positive controls re-spectively, and the VN-17L/VC-29L combinations as representatives of interaction between short-long TM segments. Additionally, the 17L and 29L homo-dimers were included to analyze the behavior of homo-topic dimers. Finally, nine more BiFC combinations (randomly selected) were also included in the assay (VN-17L/VC-25L, VN-27L/VC-27L VN-17L/VC-29L, VN-19L/VC-25L, VN-23L/VC-27L, VN-25L/VC-27L, VN-25L/VC-29L, VN-27L/VC-29L, and VN-29L/VC-23L) (**Figure 8**). As in the previous experiment, the confocal images of all tested combinations (including the highly unbalanced VN-17L/VC-29L) but those including H2 showed fluorescence levels above the negative controls. The difference in VFP signal intensity between hetero-dimers that included the 17L TM domain and any other oligomer (excluding the negative controls VN-X/VC-H2 or VN-H2/VC-X, here X being any of the tested TM segments) were also visible in the fluorescence microscope images (**Figure 8**). The fluorescence images indicate that the oligomers are located in a perinuclear region, likely the ER membrane. A correlation between fluorescence quantification via fluorescence spectrometry and confocal microscope image analysis is included in Figure S3C.

**Figure 9:**
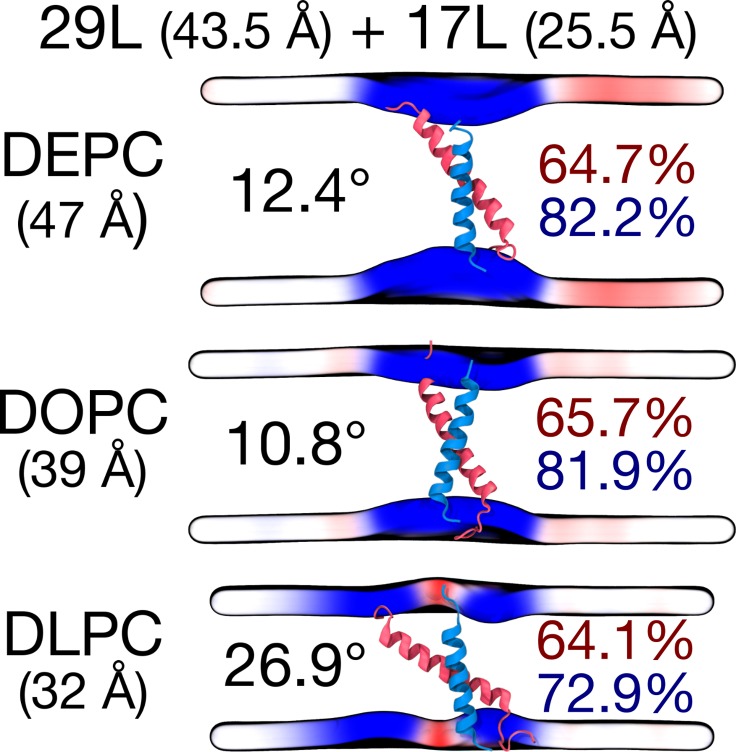
FIGURE 9: Summary of the results from MD simulations of the 17L/29L hetero-dimer. Coloring and organization as in Figure 3, except that the alpha-helical contents are given separately for the two peptides.

Computer simulations suggested that the 17L/29L hetero-dimer also remains stable and inside the membrane (Figure S1 and **Figure 3B** and **D**). The structure of the 17L peptide was stable in our simulations, while the ends of the 29L peptide fluctuated quite a lot (Figure S2). As shown in **Figure 9**, similarly to the 23L and 29L homo-dimers, the main adaptation mechanism for the 17L/29L hetero-dimer in DEPC and DOPC membranes was membrane thickness perturbation, while in the DLPC membrane the whole dimer tilted significantly maintaining the overall crossing angle between monomers. Furthermore, the alpha-helical content of the individual peptides forming the hetero-dimer was similar to their values in the corresponding homo-dimers, except for the 17L peptide of the hetero-dimer in the DLPC membrane, for which this value was somewhat lower than in the homo-dimer (**Figure 9**).

### Influence of hydrophobic length on the subcellular localization of the chimeras

**Figure 10:**
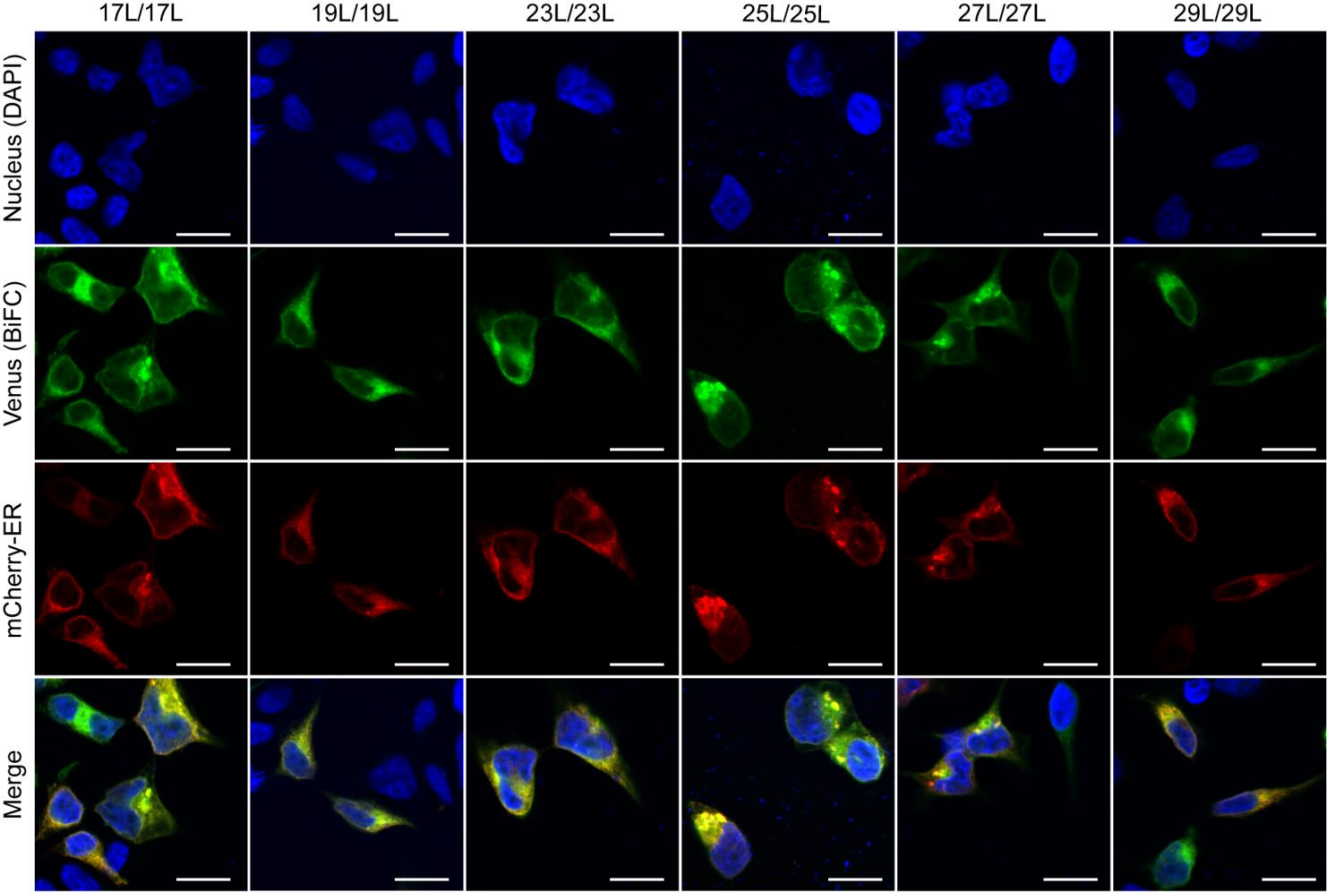
FIGURE 10: Homo-dimerization at the endoplasmic reticulum. Confocal microscopy of DAPI stained (blue) HEK293T cells expressing tested homo-dimers (17L [VN-17L/VC-17L], 19L [VN-19L/VC-19L], 23L [VN-23L/VC-23L], 25L [VN-25L/VC-25L], 27L [VN-27L/VC-27L], 29L [VN-29L/VC-29L]). TM-driven homo-oligomerization results in VFP reconstitution and fluorescent signal (green). Sec61α fused to mCherry fluorescent protein was used as ER marker (red). The presence of colocalization of red and green signals in the merge images was highlighted in yellow. Scale bar size was set to 16 μm.

**Figure 11:**
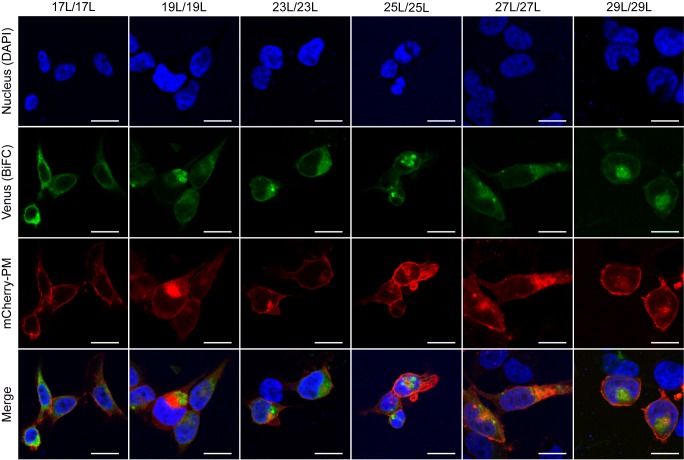
FIGURE 11: Homo-dimerization at the plasma membrane. Confocal microscopy of DAPI stained (blue) HEK293T cells expressing chimeras (17L [VN-17L/VC-17L], 19L [VN-19L/VC-19L], 23L [VN-23L/VC-23L], 25L [VN-25L/VC-25L], 27L [VN-27L/VC-27L], 29L [VN-29L/VC-29L]). TM-driven homo-oligomerization results in VFP reconstitution and fluorescent signal (green). Neuromodulin fused to mCherry fluorescent protein was used as plasma membrane marker (red). The presence of colocalization of red and green signals in the merge images was highlighted in yellow. Scale bar size was set to 16 μm.

The influence of the hydrophobic length on the subcellular localization of single spanning TM domains has been reported 48,49. However, previous confocal microscopy results (**Figure 8**) suggested a perinuclear localization of most chimeras, regardless of their hydrophobic length. To investigate the subcellular localization of the homo-oligomers we co-expressed in HEK293T cells the VN- VC- corresponding plasmids together with ER or a plasma membrane (PM) marker (**Figures 10** and **11**, respectively). The microscope-based assay revealed no considerable differences in subcellular localization among the tested TM segments and indicate that all the homo-oligomers tested, regardless of their hydrophobic length, are preferentially found in the ER membranes. Being our chimeras based on the GpA TM segment we were surprised to find them in the ER membranes and not in the plasma membrane 50. To confirm that our results were not a mere artifact we transfected HEK293T cells with a plasmid bearing the full sequence of GpA (Flag tagged) and analyzed its colocalization with ER and PM markers. The micrographs clearly indicate that the full-length GpA localizes in the PM membrane (Figure S4). Therefore, since the subcellular localization of all TM segments studied is similar we can assume that the hydrophobic match between the hydrophobic length of the TM segments and the membrane stand as a major contributor to the differences observed in our TM packing studies.

## DISCUSSION

Folding and packing of proteins depend on their sequence and the environment in which these processes take place. In the case of membrane proteins, it is the lipid bilayer that determines the type and strength of the interactions that will define the mature tertiary and/or quaternary structure. Hence, unlike the aqueous phase, cell membranes are very heterogeneous in terms of their lipid composition and organization in the membrane plane. There is a wide diversity of acyl chains and polar head groups complemented by a variety of domain structures within the bilayer plane. Thereby, membrane proteins are hosted by nanoscale environments that are dynamic and transient. In this complex scenario, the exposure of hydrophobic protein and lipid groups to water is highly unfavorable and therefore expected to be reduced. Helices in TM domains are, on average, 24 amino acids long (36 Å), ranging from 17 to 34 amino acids (25.5-51 Å) 35. A stretch of approximately 20 consecutive hydrophobic amino acids can span 30Å of the hydrocarbon core of a biological membrane. Indeed, the most prevalent (~12%) length of TM helices is 21 residues 35. Based on previous *in vitro* work, longer helices can span the bilayer with a concomitant tilting of the helix axis relative to the membrane plane 51. Other options to accommodate the wide variety of TM segments range from lipid accommodation to polypeptide backbone deformation. Alternatively, each TM segment can be located in a lipid bilayer or a lipid bilayer sub-domain that matches its hydrophobic length, thereby minimizing peptide adaptations. In fact, the subcellular distribution of helical membrane proteins among multiple organelles based on their hydrophobic lengths is considered to occur 48.

GpA offers a valuable tool for the study of hydrophobic matching in biological membranes, even in living cells. Not only successful formation of a GpA dimer depends on the correct disposition of the TM segment, but it has also been demonstrated *in vitro* that the hydrophobic mismatch influences TM helix packing in micelles 17,19. Furthermore, the NMR structure of the homo-dimer has been resolved in detergent micelles 52 and in membrane bilayers 53 and the dimerization motif has been thoroughly studied *in vitro*. Given the aforementioned characteristics of GpA, we used it to consider hydrophobic matching under *in vivo* conditions. To our knowledge, this is the first study in which hydrophobic mismatch has been systematically explored in biological membranes.

We created a set of TM segments ranging from 17 to 29 hydrophobic residues in length (25.5-43.5 Å) including the GVxxGVxxT dimerization motif. All of these segments were capable of forming homo-oligomers *in vivo*, both in prokaryotic and eukaryotic membranes. It is worth mentioning that based on our studies, each of these TM segments could oligomerize in different organelles (in eukaryotic membranes) or in different membrane domains with distinct lipid composition and hydrophobic core length. In the ToxRED assay, the presence of the GpA minimal dimerization motif turned to be not necessary for the oligomerization of long TM segments. It seems that in *E. coli* membranes the positive, but not the negative, hydrophobic mismatch drives the association of TM segments. In human-derived cells, we could observe a similar scenario. However, in this case, Gly to Ile replacement in long TM segments does have an impact on their dimerization. These results may indicate that eukaryotic membranes are more adaptable to a hydrophobic mismatch. While in HEK293T cells the membrane can adapt to a positive mismatch scenario, in *E. coli* the TM segment is mainly responsible for the elimination/reduction of the free energy associated with the exposure of hydrophobic residues to the aqueous environment.

When we assayed the formation of hetero-dimers (in eukaryotic cells using the BiFC assay) between chimeras with different hydrophobic length, we found that most of the displayed combinations returned fluorescence values significantly above the control (background) level. This result indicates that cell membranes are capable of hosting a dimer between two TM segments with large hydrophobic length disparity among them. Nonetheless, we observed significant differences in the intensity of the studied hetero-dimers. Once again, these variations could have a multi-factorial origin. Either the length of the hydrophobic segments influences partially the organelle or the membrane sub-domain localization, or the disparity in the hydrophobic length has a penalty on dimer formation, which would, for the first time, show an effect of the hydrophobic match between the TM segments and the membrane on the packing of TM domains in living cells. Based on our localization assays, the last option seems the more plausible explanation. When we studied the subcellular localization of the chimeras we found that all of them colocalized with an ER marker (**Figure 10**) but not with a PM marker (**Figure 11**). This similar localization suggests that ER membranes can adjust to the hydrophobic mismatch better than in the PM. This unexpected and homogeneous localization could also take place because dimerization precludes protein sorting, preventing the newly formed chimeras escaping from the ER. Nonetheless, our full-length GpA micrographs (Figure S4) indicate that the wild-type sequence can sort from the ER in HEK293T cells despite forming dimers. Full-length GpA sorting can probably be achieved through its native signal sequence.

Our atomistic MD simulation data indicated that 17L, 23L, and 29L homo-dimers are stable regardless of the hydrophobic thickness of the bilayer, while the dimerization motif deficient polyL control disassociates rapidly (**Figure 3**). However, the 23L and 29L peptides required some adaptation, especially in DLPC membranes where the mismatch is large and positive. The residues near the interface showed less helicity and more flexibility while keeping the structure of the dimer motif intact. Furthermore, the 23L and 29L homo-dimers in a DLPC membrane were characterized by large tilt angles. All these changes took place to keep the TM segment within the limited hydrophobic thickness of the lipid bilayers. However, no elongation of the dimer in terms of a structural change from a canonical alpha helix to the 310 helix structure was observed. Yet, a perturbation of membrane thickness near the dimer was detected. For the 17L dimer, this perturbation increased upon increasing negative mismatch (DEPC>DOPC>DLPC). For the 23L and 29L dimers, the effect was stronger or comparable for DOPC than DEPC membranes. Interestingly, in the thinner DLPC bilayer, little lipid adaptation was observed since the entire dimers tilted substantially to match the hydrophobic thickness. This suggests that there is a maximum for lipid adaptation, beyond which tilting and peptide deformation become the preferred mechanism for adaptation. The behavior of the 17L/29L hetero-dimer as a whole was similar to a 23L homo-dimer, while the structures of the individual peptides resembled those in the corresponding homo-dimers. Importantly, our simulations are able to probe degrees of mismatch larger than those appearing in the *in vivo* experiments. In the DOPC membrane, the thickness of which likely matches that of the *E. coli* and HEK293T membranes studied *in vivo*, the differences observed in dimer tilting were actually small. Therefore, the importance of this tilting in living membranes might not play a major role, and the perturbation of membrane thickness is likely the dominant adaption mechanism *in vivo*.

The stability of native GpA dimers in various lipid environments has been studied in previous works using both coarse-grained 24,25,54 and atomistic 55 simulations. These simulations reported dimerization free energies in the range of 6-10 kcal/mol. Notably, changing the membrane composition seems to have a minor effect on this value 54. Furthermore, atomistic simulations reported small differences in the dimeric structure of native GpA embedded in membranes of different thicknesses 23. Our simulation data complement these results from a novel perspective, now discussing the variation of the peptide lengths as a new variable, therefore covering larger values of mismatch and by reducing the importance of the structural features of the GpA dimer to the minimalized dimerization motif. Despite these extensions, our results agree with the earlier work on GpA.

The two adaptation mechanisms resolved here have been previously reported in systematic computational studies on KALP and WALP monomers of different lengths embedded in membranes of various thicknesses 32,33. It seems that in the case of stable dimers such as those studied here, the adaptation mechanisms are similar to the ones observed previously for peptide monomers. It must be noted that some other suggested mechanisms 33, such as the formation of a non-lamellar phase or the expulsion of the peptides from the membrane, are not feasible in our simulation study that is limited by periodic boundary conditions and achievable timescales. Furthermore, our atomistic study is limited to individual dimers. Therefore, we cannot rule out the possibility that membrane stress is relieved through the formation of larger oligomers.

Membrane proteins represent close to 30% of the human proteome 3,4,5 and 60% of the approved drug targets 56. Furthermore, processes crucial to life are regulated by elaborated interplays of membrane protein complexes, where TM domain interactions play an important role 57. In this study, we have shown and discussed pioneering data for membrane protein folding and packing in living cells, aiming to unravel the role of hydrophobic mismatch in TM helix-helix packing. Our work not only highlights the capability of biological membranes to host TM homo-oligomers with hydrophobic regions ranging from 25.5 to 43.5 Å, but it also demonstrates the ability of lipids and peptide regions to cooperate in order to minimize the hydrophobic mismatch.

## MATERIALS AND METHODS

### DNA constructs

All chimeric TM segments were obtained adding the indicated number of extra leucines to the minimized GpA dimerization motif described by 19. DNA coding for the chimeric TM segments were introduced into the ToxRED plasmid (provided by Dr. Berger) using *Xho*I and *Hind*III restriction sites (the selected restrictions sites incorporated a Lys residue preceded by a Leu residue at the amino terminus of the constructs) or into modified BiFC plasmids (pBiFC-VN155 and pBiFC-VC173, provided by Dr. Orzáez 57) fused to the amino- or carboxyl-end of the Venus Fluorescent Protein using *Not*I site. All plasmid sequences were corroborated by DNA sequencing (Macrogen, www.macrogen.com).

### ToxRED assay

The malE- *E. coli* (MM39) strain was transformed with the appropriate plasmids and plated onto Luria Bertani with 100 μg/ml ampicillin (LBA) plates. Individual colonies were inoculated into LBA medium and grown at 37°C o/n. The LBA cultures were diluted 1/100 in M9 minimal medium supplemented with 0.8% of maltose and grown at 37°C o/n. The optical density (λ595 nm) (used to normalize the fluorescence values) and the red fluorescence (λ620 nm) of the cultures expressing the chimeras were then measured using a Multimode Plate Reader Victor X3 (Perkin Elmer). For Western blots, MM39 colonies were inoculated into LBA medium and grown at 37°C until approximately OD_420_ of 0.6. At this point, cultures were harvested by centrifugation, resuspended in lysis buffer (TBS [Tris-HCl 20 mM pH7.5, NaCl 150 mM], 1% NP-40) and freeze-thaw (x3). The lysates were clarified by centrifugation (13,000 g). Supernatants were mixed with SDS-PAGE sample buffer, heated to 95°C for 5 min, separated on 12% (w/v) SDS gel and blotted onto nitrocellulose membranes. The constructs were detected with a HRP-conjugated anti-MBP monoclonal antibody (New England Biolabs) and ECL reagent (GE Healthcare). Chemiluminescence was visualized using an ImageQuant LAS 4000 (GE Healthcare) Biomolecular Imager.

### Bimolecular Fluorescence Complementation assay (BiFC)

HEK293T (ATCC) cells were cultured in Dulbecco’s modified Eagle medium (Gibco) supplemented with 10% fetal bovine serum in 24-well plates at 37°C. Plasmids encoding VN or VC fusion proteins were transfected into HEK293T using Lipofectamine 2000 transfection reagent (Invitrogen), according to manufacturer's instructions. A plasmid encoding Renilla luciferase under CMV promoter (pRL-CMV, Promega) was cotransfected for normalization purposes. For Renilla luciferase measuring, Renilla Luciferase Flash Assay Kit (Thermo Scientific) was used following the manufacturer’s instructions. The luminescence and the fluorescence of each sample were measured 48 hrs post-transfection using a Multimode Plate Reader Victor X3 (Perkin Elmer). Immuno-identification of the samples was done using α-c-Myc (VN) and α-HA tag (VC) rabbit antibodies, respectively, followed by a secondary HRP-conjugated anti-rabbit antibody (Sigma). Chemiluminescence was visualized using an ImageQuant LAS 4000 (GE Healthcare) Biomolecular Imager. P-values were adjusted to compensate for a false discovery rate (q-values) using the Benjamini and Hoechberg approach.

### Confocal fluorescence microscopy

Glass slides were treated with 30 μL of poly-L-Lysine 0.01% (Sigma-Aldrich) and washed 3 times with PBS. HEK293T cells were cultured in the glass slides and transfected as mentioned above. Cells were co-transfected with a plasmid encoding alternatively, an endoplasmatic reticulum marker (ER) (mCh-Sec61β, Addgene 49155) or a plasma membrane (PM) marker (mCh-Mem, Addgene 55779). After 2 days, cells were fixed (4% formaldehyde, Sigma-Aldrich) and DAPI stained (Fluoroshield, Sigma-Aldrich). Images were captured using a FV1000 confocal microscope (Olympus). Mean gray value of each image was measured using ImageJ (NIH).

### Immunofluorescence

HEK293T cells were transfected with a plasmid encoding full-length GpA protein with a Flag M2 tag in the amino terminus (after GpA signal peptide and cleavage site of the protease). Cells were also co-transfected with a plasmid encoding alternatively the ER or PM markers. Forty-eight hours after transfection cells were fixed (4% formaldehyde in PBS, 15 min) and washed in PBS (x3). Permeabilization was done with PBS, 1% BSA, 0.1% Triton X-100 for 2 minutes. Immuno-stainings were done using a primary α-Flag M2 antibody (Sigma), followed by a secondary Alexa647-conjugated anti-Mouse antibody (Sigma). Additionally, cells were DAPI stained for nucleus staining.

### Subcellular fractionation

HEK293T cells were transfected with plasmids encoding VN fusion proteins and cultured for 48h as mentioned above. As a soluble protein marker, we used the Enhanced Yellow Fluorescent Protein (EYFP) bearing a Flag M2 tag. Cells were collected with 500 μL of a subcellular fractionation (SF) buffer (250mM Sucrose, 20 mM HEPES pH 7.4, 10 mM KCl, 1.5 mM MgCl_2_, 1 mM EDTA, 1 mM EGTA) and lysed by sonication. Lysates were incubated at 4°C for 30 min and centrifuged (10,000 g, 4°C, 10 min). Supernatants were carefully transferred to a new tube and ultracentrifuged at 10,0000g at 4°C for 1 h. The supernatants of the ultracentrifugation (cytosolic fraction) were stored while the pellet was re-suspended in 500 μL of SF buffer and ultracentrifuged again using the same conditions. The final pellet (membrane fraction) was re-suspended in 150 μL of 1x SDS-PAGE buffer.

### Structural models of the simulated system

The structures of three peptide homo-dimers (17L, 23L, and 29L), one hetero-dimer (17L/29L), and a polyleucine control (corresponding to the 23L dimer without the dimerization motifs, PolyL) were prepared using the GpA dimer (PDB:1AFO, 52) as a template. Using MODELLER 58, the structures of the dimers were aligned with the dimeric GpA NMR structure. The required mutations and/or the extension of the peptide were then performed. A total of 100 models were created and the ones with the best match to GpA were chosen to preserve the structure of the dimerization motif. The C-termini of these most appropriate models were left charged, while the N-termini were acetylated to mimic the chimeric polypeptides studied in cells. The dimers were embedded in three lipid bilayers of varying thickness using CHARMM-GUI 59: 1,2-dilauroyl-sn-glycero-3-phosphocholine (DLPC, di-12:0), 1,2-dioleoyl-sn-glycero-3-phosphocholine (DOPC, di-18:1), or 1,2-dierucoyl-sn-glycero-3-phosphocholine (DEPC, di-22:1). The polyleucine control (dimerization motif-less) was only studied in DOPC. These bilayers consisted of a total of 400 lipids (200 per leaflet), and they were solvated by a total of 24,000 water molecules and 134 mM of NaCl.

### Atomistic molecular dynamics simulations

The peptides 60 and the lipids 61 were modeled using the CHARMM36 force field, while the CHARMM-specific TIP3P model was employed for water. The systems were equilibrated following the standard protocol of CHARMM-GUI, in which the position restraints are stepwise reduced. For each of the systems, this was followed by 1 microsecond of unbiased simulation, where the last 500 ns whenever averages of quantities were calculated. The polyleucine control was only simulated for 500 ns. These simulations were performed in the NPT ensemble using a leap-frog integrator and a time step of 2 fs. The pressure was maintained semi-isotropically at 1 bar using the Parrinello-Rahman barostat 62 with a coupling time constant of 5 ps and a compressibility of 4.5x10^-5^ bar^-1^. The temperatures of the peptides, lipids, and solvent were separately maintained at 37°C using the Nosé-Hoover thermostat 63 with a time constant of 1 ps. The smooth particle mesh Ewald algorithm was employed for electrostatics 64. The Lennard-Jones interactions were (force-)switched to zero between 10 and 12 Å. The buffered Verlet list cut-off scheme was employed 65. The lengths of bonds involving hydrogen atoms were constrained using LINCS 66. Trajectories were written every 100 ps for analysis. All simulations were performed using Gromacs v. 5.1.x 67. All our simulation data are openly available online for everyone here and here with a movie of the simulations available here.

### Analyses of simulation data

Root mean squared deviation (RMSD) and root mean square fluctuation (RMSF) were analyzed using the Gromacs tools gmx rms and gmx rmsf. Dimer tilt angles were evaluated using the gmx bundle tool. Two angles were used to describe the orientation of the peptides within the membrane, both were evaluated using the gmx bundle tool. The dimer tilt angle, describing the tilt of the dimer as a whole, was calculated as follows: the center of mass (COM) of the three subsequent leucine residues right above the dimerization motifs in the two peptides defined the top of the dimerization motif. The bottom of the motif was defined similarly using the COM of the three leucine residues right below the motifs in the two peptides. The tilt angle of the dimer was defined as the angle between a vector connecting the top and the bottom of the motif and the z axis (normal to the bilayer). The crossing angle of the dimer, describing the mutual orientation of the two peptides, is defined as the angle between the two vectors connecting the leucine residues right next to the dimerization motif in the individual peptides. The alpha-helical content of the peptides was evaluated using the gmx do_dssp and DSSP 68 tools. The thickness maps were calculated using g_lomepro 69. Membrane thickness was defined as the inter-leaflet phosphorus-phosphorus distance, and membrane hydrophobic thickness as the inter-leaflet distance between the fatty acid chain alpha carbons. Both thicknesses were analyzed employing a grid spacing of 0.33 nm, and the figures displaying the surfaces formed by the phosphorus atoms were rendered using VMD 70. The thickness values of the membrane bulk were estimated as the average thickness of the membrane at the grid points furthest away from the protein.

## SUPPLEMENTAL MATERIAL

Click here for supplemental data file.

All supplemental data for this article are also available online at http://www.cell-stress.com/researcharticles/the-role-of-hydrophobic-matching-on-transmembrane-helix-packing-in-cells/.

## Abbreviations

BiFC: Bimolecular Fluorescent Complementation
ER: endoplasmic recticulum
GpA: Glycophorin A
MBP: maltose binding protein
RFP: red fluorescent protein
TM: transmembrane
VFP: venus fluorescent protein
