# Supplementary material for: The role of hydrophobic matching on transmembrane helix packing in cells
Source: Cell Stress. 2017 Nov 2;1(2):90–106. doi: 10.15698/cst2017.11.111 (PMC6551820; doi:10.15698/cst2017.11.111)
Supplement: Supplementary file 1 [file ces-01-090-s01.pdf]

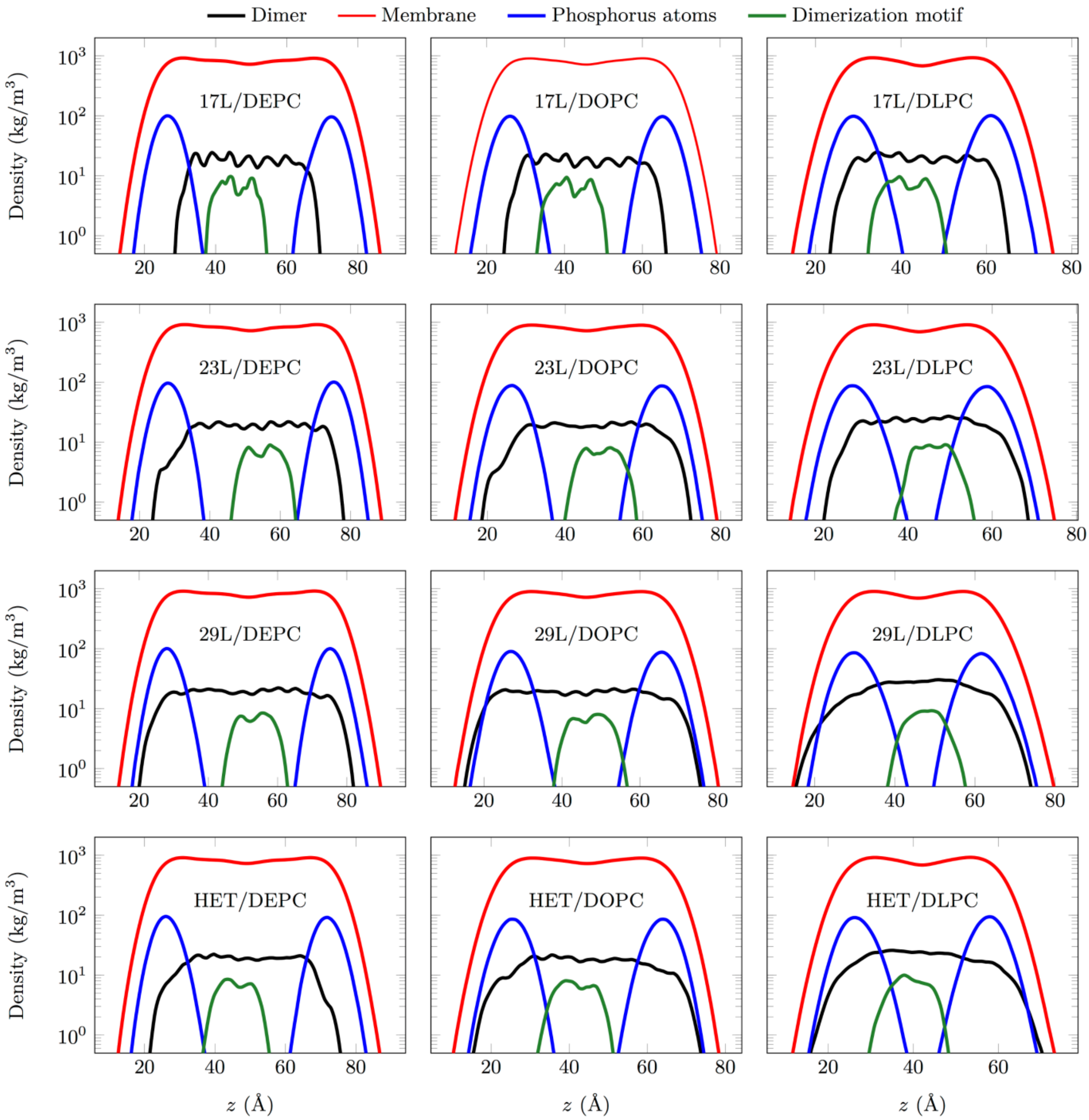

Supplemental FIGURE 1: Partial mass density profiles along the direction normal to the membrane for selected components in the atomistic simulations. All peptides remain positioned in the bilayer, and the location of the dimerization motif is always buried deep in the hydrophobic core. The 17L/29L hetero-dimer is labeled "HET".

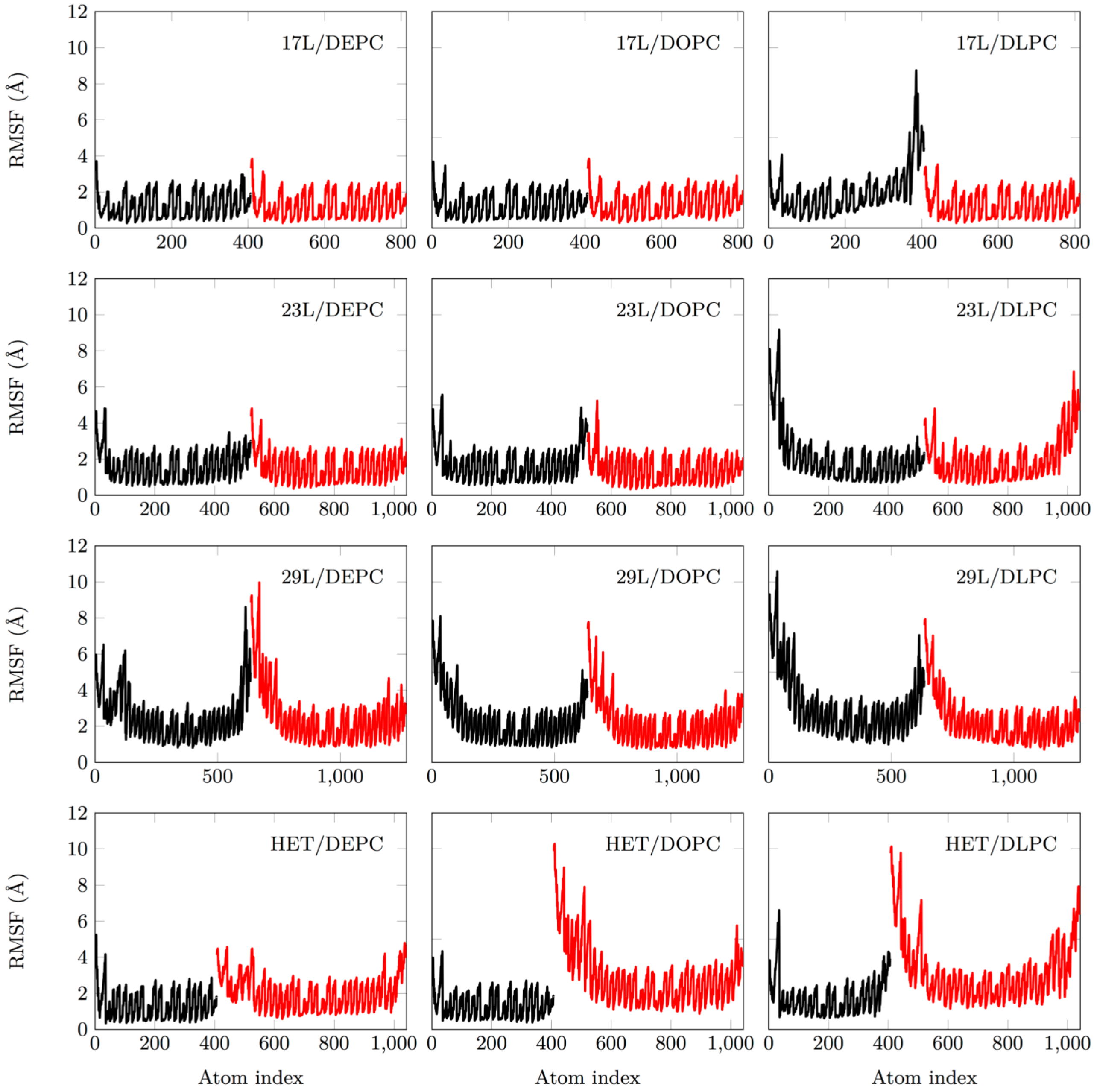

Supplemental FIGURE 2: Root mean squared fluctuations (RMSF) of the peptide atoms during the last 500 ns of the simulations. The two peptides are shown in black and red. The 17L/29L hetero-dimer is labeled "HET".

A

VC

VN	17L	19L	23L	25L	27L	29L
17L	2.12	1.72	1.77	1.55	1.60	1.60
19L	1.29	2.25	2.17	1.83	1.90	1.42
23L	1.81	1.92	2.25	1.76	2-15	2.08
25L	1.56	2.07	2.36	2.22	2.04	1.94
27L	1.66	1.82	2.46	1.82	2.62	2.18
29L	1.79	1.98	2.25	1.92	2.28	2.20

q-value

>0.05	<0.05

B

VC

VN	17L	19L	23L	25L	27L	29L
17L	1.00 n/a	0.79	0.81	0.72	0.68	0.74
19L	0.59	1.00 n/a	0.96	0.82	0.79	0.71
23L	0.83	0.85	1.00 n/a	0.79	0.89	0.94
25L	0.72	0.93	1.06	1.00 n/a	0.85	0.88
27L	0.71	0.75	1.01	0.76	1.00 n/a	0.91
29L	0.83	0.89	1.02	0.87	0.95	1.00 n/a

q-value

Homo	>0.05	<0.05 lower	<0.05 equivalent

C

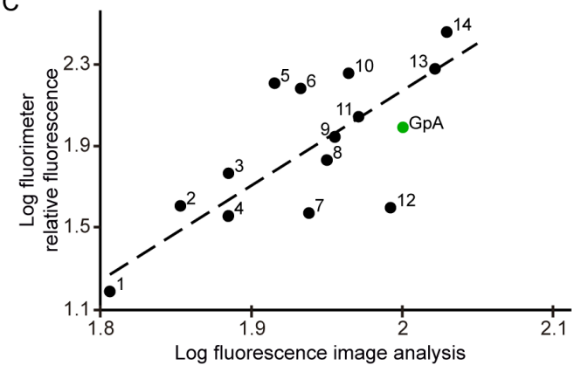

Supplemental FIGURE 3: Panels (A) and (B) Heat map representation of BiFC hetero-oligomerization assay. Values represented as bar graphs in Figure 6 and 7. **(C)** Comparison of chimera's VFP reconstitution values obtained via fluorimeter or confocal images quantification. The dots correspond to the combinations included in Figure 8 (1 (VN-17L/VC-H2), 2 (VN-17L/VC-29L), 3 (VN-17L/VC-25L), 4 (VN-23L/VC-25L), 5 (VN-29L/VC-29L), 6 (VN-27L/VC-29L), 7 (VN-29L/VC-H2), 8 (VN-19L/VC-25L), 9 (VN-25L/VC-29L), 10 (VN-29L/VC-23L), 11 (VN-25L/VC-27L), 12 (VN-17L/VC-27L), 13 (VN-17L/VC-17L), 14 (VN-27L/VC-23L)). GpA was highlighted in green (not included in the trend line analysis).).

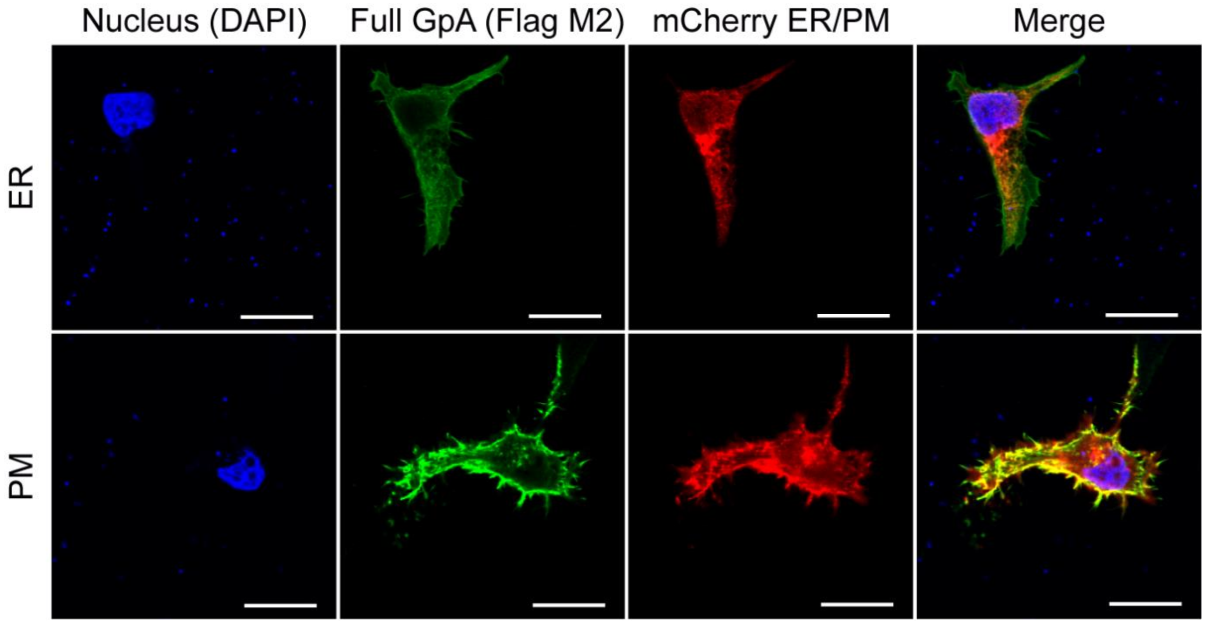

Supplemental FIGURE 4: Sub-cellular localization of GpA. Confocal microscopy of DAPI stained (blue) HEK293T cells expressing full length GpA protein (Flag-tagged). Neuromodulin fused to mCherry fluorescent protein was used as a plasma membrane marker (PM) while Sec61α fused to mCherry fluorescent protein was used as endoplasmic reticulum marker (ER).
